# Outcomes of Hospitalized Acute Alcoholic Hepatitis (AH) in Patients With Bipolar 1 Disorder (B1D)

**DOI:** 10.7759/cureus.25418

**Published:** 2022-05-27

**Authors:** Alexander J Kaye, Shivani Patel, Sarah Meyers, Daniel Rim, Catherine Choi, Sushil Ahlawat

**Affiliations:** 1 Internal Medicine, Rutgers University New Jersey Medical School, Newark, USA; 2 Psychiatry, Rutgers Robert Wood Johnson Medical School, Piscataway, USA; 3 Gastroenterology and Hepatology, Rutgers University New Jersey Medical School, Newark, USA

**Keywords:** hepatotoxicity, alcohol use disorder, acute hepatic failure, bipolar disorder, alcoholic hepatitis

## Abstract

Introduction: Alcoholic hepatitis (AH) is a common cause of hospital admissions and is associated with a high mortality rate. AH occurs frequently in patients with heavy alcohol use. Alcohol use disorder (AUD) commonly presents with comorbid psychiatric disorders such as bipolar disorder. Bipolar disorder patients are also known to be at an increased risk for chronic liver diseases. Bipolar 1 disorder (B1D) is often considered the most severe presentation among different types of bipolar disorder. This study assesses the clinical outcomes of patients admitted for AH with concomitant B1D.

Methods: Adult patients with AH were identified within the 2014 National Inpatient Sample (NIS) database. International Classification of Diseases, Ninth Edition Revision, Clinical Modification (ICD-9 CM) codes were used to select for all of the diagnoses for this study. AH patients were subdivided into those with and without B1D. The outcomes of interest were sepsis, hepatic encephalopathy, acute respiratory failure, acute kidney injury, ischemic stroke, hepatic failure, coagulopathy, and inpatient mortality. A multivariate logistic regression analysis was performed to explore whether B1D is an independent predictor for the outcomes.

Results: Among 4,453 patients with AH identified, 166 patients also had B1D. AH patients with comorbid B1D were seen to be younger (42.9 years old vs. 46.2 years old, p < 0.05) and more commonly female (55.4% vs. 36.5%, p < 0.05). The B1D subgroup of AH patients were found to less likely develop acute hepatic failure (adjusted odds ratio (aOR) 0.13, 95% confidence interval (CI): 0.02-0.97, p < 0.05). The adjusted odds ratios for the remaining outcomes were not statistically significant.

Conclusions: Our study indicates that B1D may be an independent protective factor against acute hepatic failure in patients hospitalized with AH. This finding can be explained by frequent laboratory monitoring and psychiatric assessments performed by psychiatrists treating B1D patients, as well as the impact B1D has on cortisol release induced by hyperactivity of the hypothalamic-pituitary-adrenal (HPA) axis.

## Introduction

Alcohol use disorder (AUD) is a chronic disease with a prevalence of approximately seven percent among adults within USA [1]. Chronic alcohol use can result in liver disease via oxidative damage and injury to hepatocytes [2]. In the setting of excessive alcohol use, a possible complication is acute hepatic inflammation with jaundice, also termed alcoholic hepatitis (AH) [3]. While the exact incidence of AH among patients with AUD is unknown, based on histologic evidence, it is thought to occur in 10% to 35% of patients with heavy alcohol use [4]. Risk factors for AH include high quantity of alcohol intake, an older age, being male, malnutrition, genetic polymorphisms such as the patatin-like phospholipase domain-containing protein 3, obesity, and other comorbid hepatic pathologies [1,5].

Overall, AH has a 30% to 50% mortality rate within three months and an annual incidence of 10% to 20% for progression to cirrhosis [1,5]. The diagnosis of AH can be difficult, but the criteria include alcohol use within 60 days, presence of elevated liver enzymes, jaundice, and absence of other causes for the presentation [2]. The mainstay of treatment is supportive management for mild to moderate AH [3]. Standardized scoring systems such as Maddrey’s Discriminant Function score and Glasgow Alcoholic Hepatitis score can be utilized to assess the indication for glucocorticoids in the setting of severe AH [1].

One out of every three AUD patients presents with a comorbid psychiatric diagnosis [6]. In particular, bipolar disorder has been found to have a strong association with AUD, with 32% of bipolar disorder patients also diagnosed with AUD [6]. Bipolar disorder encompass a range of presentations including bipolar 1 disorder (B1D), bipolar 2 disorder, and cyclothymic disorders [7]. In particular, B1D, often considered the most severe presentation, includes both manic and depressive episodes, and at times psychosis [8]. Among the spectrum of bipolar disorder, B1D patients are at the highest risk for comorbid AUD; approximately 46% of all B1D patients are also diagnosed with AUD [9].

This high co-occurrence of bipolar disorder and AUD is associated with a range of negative consequences including a higher likelihood of medication noncompliance, slower recovery from a mood episode, an increased frequency of hospitalizations, suicidal behavior and accidents [10]. In addition, patients with bipolar disorder are also found to be at higher risk for chronic liver disease with alcohol use and hepatotoxic mood stabilizers as notable contributing risk factors [11]. More specifically, patients with bipolar disorder in a prior study were found to more likely develop hepatic steatosis, alcoholic cirrhosis, and non-alcoholic cirrhosis [11]. Despite the established associations between AUD and AH, as well as between AUD, chronic liver disease and bipolar disorder, there is limited research exploring the direct relationship of AH and bipolar disorder. This study explores the outcomes of patients hospitalized with AUD with a history of comorbid B1D. An abstract of this study was presented at the ACG 2021 Annual Meeting in October 2021 in Las Vegas, USA.

## Materials and methods

This research was conducted as a retrospective cohort study of adults (at least 18 years old) hospitalized for AH during the 2014 year. The patients were selected from the National Inpatient Sample (NIS), Healthcare Cost and Utilization Project (HCUP), and the Agency for Healthcare Research and Quality, which is known as the largest all-payer inpatient database in USA [12]. The diagnoses and outcomes assessed in this study were identified from the NIS database utilizing International Classification of Diseases, Ninth Edition Revision, Clinical Modification (ICD-9 CM) codes. The patients admitted with AH were then divided between a subgroup with and a subgroup without comorbid B1D. Demographic and hospitalization information such as age, sex, race, length of stay, hospitalization cost, and chronic liver diseases was also obtained from the NIS database for each of the subgroups and subsequently compared. The Charlson Comorbidity Index, a standardized calculation that assesses a 10-year mortality risk based on multiple potential comorbidities, was formulated for each of the subgroups and subsequently compared [13,14].

The Statistical Package for the Social Sciences (SPSS) version 28.0.0 (IBM corporation, Armonk, NY) was used for all statistical analyses performed in this study. The outcomes of interest in the study were sepsis, hepatic encephalopathy, acute respiratory failure, acute kidney injury, ischemic stroke, acute hepatic failure, coagulopathy, and inpatient mortality. Chi-squared tests and independent T-tests were used to compare proportions and means respectively. Statistical analyses performed were two-tailed, and a p-value threshold of less than 0.05 was deemed statistically significant. The categorical variables were reported as numbers (N) and percentages (%), and continuous variables were reported as means ± SD. A multivariate logistic regression analysis was also completed to identify whether B1D is an independent predictor for the aforementioned clinical outcomes, after adjusting for the age, sex race, and Charlson Comorbidity Index.

## Results

Among 4,453 total patients admitted for AH, 166 patients had comorbid B1D. As exhibited in Table 1, the patients in the B1D subgroup were younger (42.9 years old vs. 46.2 years old, p < 0.05) and more likely to be female (55.4% vs. 36.5%, p < 0.05). There was no statistically significant difference in race (p = 0.90), length of stay (p = 0.58), total hospital charge (p = 0.99), Charlson Comorbidity Index (p = 0.06), alcoholic fatty liver (p = 0.25), and alcoholic cirrhosis (p = 0.12).

**Table 1:** Demographics, characteristics, length of stay, total hospital charge, and Charles Comorbidity Index among alcoholic hepatitis (AH) patients with and without a history of bipolar 1 disorder (B1D) *The sample size is less than 10, which is not permitted to be reported by National Inpatient Sample (NIS), Healthcare Cost and Utilization Project (HCUP), and the Agency for Healthcare Research and Quality.

Variable	With B1D	Without B1D	p-value
N = 4,453	N = 166	N = 4,287	
Patient age, mean (SD)	42.9 (10.1)	46.2 (11.5)	< 0.05
Sex, N (%)			< 0.05
Female	92 (55.4%)	1566 (36.5%)	
Male	74 (44.6%)	2720 (63.5%)	
Race, N (%)			0.90
White	113 (72.9%)	2872 (71.6%)	
Black	19 (12.3%)	415 (10.3%)	
Hispanic	15 (9.7%)	495 (12.3%)	
Asian or Pacific Islander	*	52 (1.3%)	
Native American	*	70 (1.7%)	
Other	*	109 (2.7%)	
Length of stay, in days (SD)	5.7 (5.7)	5.4 (6.0)	0.58
Total hospital charges, in $ (SD)	40,205 (62,169)	40,296 (81,193)	0.99
Charlson Comorbidity Index, mean (SD)	0.79 (1.15)	0.97 (1.22)	0.06
Chronic liver diseases, N (%)			
Alcoholic fatty liver	10 (6.0%)	366 (8.5%)	0.25
Alcoholic cirrhosis	33 (19.9%)	1,080 (25.2%)	0.12

After adjusting for age, sex, race, and Charlson Comorbidity Index, B1D patients were found to less likely have acute hepatic failure (adjusted odds ratio (aOR) 0.13, 95% confidence interval (CI): 0.02-0.97, p < 0.05) as displayed in Table 2. However, the adjusted odds ratios for sepsis (aOR 0.71, 95% CI: 0.31-1.64, p = 0.42), hepatic encephalopathy (aOR 1.36, 95% CI: 0.86-2.13, p = 0.19), acute respiratory failure (aOR 0.63, 95% CI: 0.23-1.73, p = 0.37), acute kidney injury (aOR 0.80, 95% CI: 0.47-1.36, p = 0.41), ischemic stroke (aOR 7.21, 95% CI: 0.74-70.44, p = 0.09), coagulopathy (aOR 1.17, 95% CI: 0.14-10.11, p = 0.89), and inpatient mortality (aOR 0.78, 95% CI: 0.24-2.51, p = 0.68) did not meet the cut off for statistical significance.

**Table 2:** Multivariate logistic regression analysis of clinical outcomes among alcoholic hepatitis (AH) patients with and without a history of bipolar 1 disorder (B1D) *Adjusted for age, sex, race, and the Charlson Comorbidity Index.

Outcomes	Adjusted odds ratio*	95% Confidence Interval	p-value
Sepsis	0.71	0.31-1.64	0.42
Hepatic encephalopathy	1.36	0.86-2.13	0.19
Acute respiratory failure	0.63	0.23-1.73	0.37
Acute kidney injury	0.8	0.47-1.36	0.41
Ischemic stroke	7.21	0.74-70.44	0.09
Acute hepatic failure	0.13	0.02-0.97	<0.05
Coagulopathy	1.17	0.14-10.11	0.89
Inpatient mortality	0.78	0.24-2.51	0.68

## Discussion

This study found that those diagnosed with AH and B1D were more likely to be female despite prior literature suggesting that men are at higher risk of both AUD and B1D [15]. This may be a consequence of the sex differences in the amount and frequency of alcohol consumption leading to liver disease. While men are more likely to develop AUD, a psychiatric diagnosis based on a series of criteria that collectively indicate a problematic pattern of alcohol that results in significant distress or impairment, women are more likely to experience alcoholism [16,17]. In patients with bipolar disorder, the odds ratio for developing alcoholism is 7.53 in women compared to 2.77 in men [17]. In addition, a woman’s’ risk of liver disease increases with consumption of 20 to 40 g of alcohol per day [1]. This is in contrast to men who generally need to consume between 60 and 80 g of alcohol per day to be at an equivalent risk for liver disease [1]. Thus, women may be more likely to suffer from liver injury with equivalent alcohol consumption.

Notably, this study also found that B1D is protective against acute hepatic failure in patients who are admitted with AH with an adjusted odds ratio of 0.13. At first, this finding may seem unexpected given most pharmacotherapies for bipolar disorder can theoretically cause hepatotoxicity [18]. Mood stabilizing pharmacologic agents mainly fall into one of three groups, those being lithium, antiepileptic medications like carbamazepine and valproic acid, and antipsychotics such as olanzapine, quetiapine, and risperidone [18,19]. While lithium can cause hyperbilirubinemia in rare situations, since it is primarily renally excreted, it is one of the few mood stabilizing agents that is not considered hepatotoxic, unlike antiepileptic and antipsychotic medications [19]. B1D often requires lifelong pharmacologic maintenance therapy to maintain the psychiatric stability of the patient, however this subsequently entails regular serologic monitoring every three to 12 months based on the medication utilized and how long the patient has been using a given mood stabilizing agent [19,20]. In the beginning of therapy, serologic testing of liver function enzymes may be as frequent as every month for agents such as carbamazepine, and valproic acid [21]. This frequency of serologic testing by psychiatrists may contribute to an earlier diagnosis of AH, thereby allowing earlier management than those without B1D.

In addition to close laboratory monitoring, psychiatrists are well-positioned to regularly screen and treat AUD in bipolar disorder patients. When undergoing psychiatric evaluation, screening for substance use and medical illness that may impact psychiatric treatment is considered the standard of care [22]. In particular, alcohol screening is considered essential in bipolar disorder patients given the high prevalence of AUD and psychoactive drug use more generally [23]. The frequency of comorbid bipolar disorder and AUD has necessitated psychiatrists to prepare integrated plans of care. Examples of integrated strategies used by psychiatrists to treat both diagnoses include psychotherapy, group therapy, early recovery adherence therapy, and medication management [24,25]. Through screening and treatment of AUD by psychiatrists, B1D patients may be further protected from severe AH that could progress to acute hepatic failure.

Another possible explanation for the results of this study may relate to the impact bipolar disorder has on the hypothalamic-pituitary-adrenal (HPA) axis. Patients with bipolar disorder have increased cortisol levels and increased HPA axis activity [26]. Patients with AH have been found to experience adrenal insufficiency that worsens with the severity of the AH [27]. Given the hyperactivity of the HPA axis in the bipolar disorder population, this may help mitigate the adrenal insufficiency that non-bipolar disorder patients with AH experience. In patients with severe AH, use of corticosteroids have demonstrated improved clinical outcomes [28]. Thus, the elevated cortisol levels in bipolar disorder patients may mitigate the risk of progression to acute hepatic failure.

There were several notable limitations of this research. A key limitation relates to the functionality of database research. The NIS database relies on accurate billing by healthcare providers. Lack of precise billing can lead to the over or underrepresentation of the B1D subgroup of patients admitted with AH in addition to the outcomes evaluated in this study. In particular, bipolar disorder is commonly misdiagnosed as unipolar depression, which consequentially reduces the number of B1D patients found to be admitted with AH [29]. Another limitation of this study was its inability to assess the severity of AH or whether the patient was treated with corticosteroids. This is a result of the lack of specific ICD-9 billing codes to help define disease severity or treatment modality for AH. Despite the aforementioned limitations, a significant strength of the study is the ability to assess patient demographics and outcomes of AH on a national scale. In addition, this study was strengthened by the use of a multivariate logistic regression analysis that adjusted for numerous potential confounding variables.

## Conclusions

In conclusion, this study demonstrated that B1D may be an independent protective factor against acute hepatic failure in patients with AH. This protective effect is possibly due to the increased frequency of serologic testing and psychiatric screening performed by psychiatrists. Psychiatrists are well-positioned to identify early laboratory abnormalities and treat AUD during their maintenance care of B1D patients. Possibly through modeling after the psychiatric approach to comorbid bipolar disorder and AUD, Internal Medicine and Gastroenterology and Hepatology may be able to play a larger role in helping reduce the number of patients who are admitted with AH and the number of severe AH presentations that occur. In addition, given the hyperactivity of the HPA axis in bipolar disorder patients and the role this may play in reducing acute hepatic failure in AH, further research is warranted to explore the potential benefit of more frequent adrenal insufficiency screening, and subsequent treatment with corticosteroids.
